# Supplementary material for: Study on the Changes and Correlation of Microorganisms and Flavor in Different Processing Stages of Mianning Ham
Source: Foods. 2024 Aug 18;13(16):2587. doi: 10.3390/foods13162587 (PMC11353924; doi:10.3390/foods13162587)
Supplement: Supplementary file 1 [file foods-13-02587-s001.zip › foods-3152162-supplementary.pdf]

Table S1. Contents of volatile flavor substances in Mianning ham at different processing stages

Compounds	Name	Absolute Content (μg/kg)								
		HT1	HT2	HT3	HT4	HT5	HT6	HT7	HT8	HT9
						Aldehydes（41）				
1	(E)-2-methyl-2-butenal	-	-	-	-	-	-	-	6.58±1.22	-
2	sec-Butyl nitrite	-	-	-	-	-	6.28±0.53	5.35±0.27	-	-
3	Hexanal	33.61±8.67	3073.98±907.00	3448.82±629.17	5036.25±872.00	2589.74±294.30	1359.98±191.36	866.16±78.84	2527.49±197.48	837.17±153.65
4	(E)-2-hexenal	-	-	30.60±0.96	-	-	-	-	-	-
5	3-Methyl-3-cyclohexen-1-carboxaldehyde	-	-	52.16±6.21	-	-	-	-	-	-
6	Heptanal	6.85±1.55	342.08±141.67	329.47±29.91	471.75±71.32	224.69±13.45	187.99±26.58	247.80±25.93	330.98±32.42	92.66±19.27
7	Methional	-	-	-	-	-	-	21.01±5.04	32.30±4.69	13.36±2.95
8	Benzaldehyde	6.64±1.70	-	-	-	-	76.59±6.66	216.26±103.42	203.60±26.29	57.94±14.91
9	(Z)-2-heptenal	-	-	312.15±8.15	-	-	-	-	-	-
10	Octanal	14.80±2.25	541.19±169.18	380.54±53.12	699.21±139.57	318.89±13.31	399.53±62.42	553.56±81.9	541.10±69.94	126.81±26.70
11	Benzeneacetaldehyde	-	-	-	-	-	35.72±4.62	133.58±12.74	310.71±107.76	113.49±24.50
12	(E)-2-octenal	-	206.93±46.46	623.18±18.86	868.51±115.35	389.20±24.22	147.05±22.30	71.23±39.41	264.77±22.20	70.21±17.62
13	Nonanal	39.46±9.19	1413.92±426.34	965.55±70.41	1672.05±313.64	1073.08±108.35	1285.19±187.92	1621.51±307.89	1340.60±148.38	354.39±78.06
14	3-Benzyloxy-2-fluoro-4-methoxy-benzaldehyde	-	-	-	-	-	-	5.97±1.64	-	-
15	5-Heptyldihydro-2(3H)-furanone	-	-	-	-	-	-	-	-	3.32±0.24
16	4-Ethyl-benzaldehyde	-	-	-	-	-	19.89±1.93	12.31±1.83	47.11±7.26	47.17±3.72
17	(E)-2-Nonenal	-	102.51±20.89	241.19±10.93	447.38±63.61	175.49±21.05	111.00±12.84	99.13±22.70	214.23±20.97	50.75±9.62
18	(E)-2-Tridecenal	-	-	-	-	-	-	-	-	1.66±0.28
19	cis-4-Decenal	-	35.23±2.29	-	-	-	32.79±1.14	-	-	-

Compounds	Name	Absolute Content (μg/kg)								
		HT1	HT2	HT3	HT4	HT5	HT6	HT7	HT8	HT9
20	(E)-4-Decenal	-	53.92±2.73	-	-	-	-	-	-	-
21	Decanal	-	50.19±15.66	38.58±5.65	73.24±26.07	53.71±6.75	80.85±14.08	286.60±118.95	107.12±9.21	50.25±9.22
22	(E, E)-2, 4-Nonadienal	-	-	72.03±2.79	109.81±17.20	39.81±5.07	23.38±3.47	12.76±1.04	51.15±5.66	8.22±0.61
23	(E)-4-Nonenal	-	-	-	-	-	7.67±0.34	4.11±0.80	16.28±0.57	4.98±0.20
24	cis-4-Decenal	-	-	-	-	-	7.93±0.36	8.34±0.20	12.17±1.24	6.98±0.71
25	2-Dodecenal	-	-	-	-	89.52±4.01	-	-	-	-
26	(E)-2-Decenal	-	183.20±37.00	380.58±18.41	634.29±79.76	163.12±16.88	210.26±36.43	219.27±57.81	375.80±54.10	77.50±1.27
27	(E, E)-2, 4-Dodecadienal	-	-	-	-	-	24.77±6.53	30.76±3.09	47.26±4.19	49.27±3.86
28	2, 4-Dodecadienal	-	-	-	-	-	22.88±2.00	18.02±2.93	15.75±2.11	10.67±1.98
29	Undecanal	-	-	-	-	-	11.53±0.48	25.54±2.52	18.20±2.64	4.05±4.00
30	(E, E)-2, 4-Decadienal	-	-	51.77±2.27	110.97±17.12	40.77±7.80	36.25±7.28	30.17±7.07	116.63±13.71	11.39±7.21
31	Apricolin	-	-	-	-	-	14.45±0.56	20.4±3.70	35.33±7.34	36.58±16.55
32	2-Undecenal	-	138.95±34.82	201.15±19.66	363.11±49.33	269.28±273.29	185.20±31.13	169.68±37.74	276.40±42.68	200.28±32.87
33	Dodecanal	-	-	-	41.37±1.61	-	14.27±1.54	32.86±1.59	11.91±1.64	12.40±0.21
34	(E)-2-Dodecenal	-	-	-	-	-	-	3.75±0.09	-	-
35	4-Pentyl-benzaldehyde	-	-	-	-	-	7.28±0.42	4.50±0.30	5.73±1.08	5.27±0.26
36	Tridecanal	-	-	-	-	-	10.25±0.35	-	-	-
37	Tetradecanal	-	41.61±0.54	-	-	-	21.82±1.96	10.92±0.49	18.27±0.26	24.69±1.12
38	Pentadecanal	-	40.82±1.62	-	-	-	42.21±5.66	20.54±2.35	12.10±1.96	8.27±1.04
39	Hexadecanal	-	-	-	-	59.84±50.70	301.82±26.79	221.86±31.53	162.09±6.55	26.75±2.19
40	(Z)-13-Octadecenal	-	-	-	-	-	3.32±0.32	-	-	-
41	10-Undecenal	-	-	-	-	-	4.91±0.68	3.47±0.24	-	-
					Ketones（25）					
1	2-Hexanone	-	-	-	-	-	-	4.30±0.20	-	-

Compounds	Name	Absolute Content (μg/kg)								
		HT1	HT2	HT3	HT4	HT5	HT6	HT7	HT8	HT9
2	Cis-hexahydro-1, 3-benzodioxol-2-one	-	-	-	-	-	3.43±0.96	5.36±0.26	7.45±0.28	-
3	2-Heptanone	-	-	-	-	-	70.13±6.47	121.35±10.48	-	-
4	5-Methyl-2-hexanone	-	76.27±32.13	56.99±0.53	65.58±4.50	52.48±3.58	73.00±2.25	80.33±1.38	87.41±5.78	43.54±10.61
5	3-Methyl-1, 4, 6, 7-tetrahydro-pyrazolo [3, 4-c] pyridin-5-one	-	-	-	-	-	-	-	-	5.58±2.08
6	(Z)-3-hepten-2-one	-	-	-	-	-	-	-	-	1.68±0.10
7	5-Methyl-3-heptanone	-	-	-	-	-	-	-	17.60±0.90	-
8	3,5-Octadien-2-one	-	-	-	-	69.34±3.45	-	-	112.80±18.80	-
9	2-Nonanone	-	-	-	-	-	-	202.55±23.65	-	-
10	1-(2, 2-Dimethylcyclopentyl)-ethanone	-	-	-	-	-	-	-	50.62±11.42	3.35±0.21
11	3, 6-Dimethyl-octan-2-one	-	-	-	-	-	10.82±0.23	29.81±5.73	34.70±8.30	43.53±14.03
12	1-(2, 4-Dimethyl-furan-3-yl)-ethanone	-	-	-	-	-	-	22.75±1.02	-	-
13	1, 4-Cyclooctanedione	-	-	45.66±2.73	62.05±3.44	-	-	-	-	-
14	2-(3-Methyl-2-buten-1-yl)-cyclopentanone	-	-	-	-	-	-	-	-	3.07±0.46
15	Adrenalone	-	-	-	-	-	-	5.55±0.04	-	-
16	Pulegone	-	-	-	-	-	-	4.26±0.24	-	-
17	4, 7, 7-Trimethyl-3, 9-	-	-	45.93±3.70	-	-	-	-	-	-

Compounds	Name	Absolute Content (μg/kg)								
		HT1	HT2	HT3	HT4	HT5	HT6	HT7	HT8	HT9
	dioxatricyclo [6.1.0.0(2,4)] nonan-5-one									
18	Cyclodecanone	-	-	-	-	27.8±1.92	18.65±2.32	9.96±6.42	24.68±3.06	25.47±2.79
19	1-Methyl-2-pyrrolidinethione	-	-	-	-	-	-	-	-	4.91±0.23
20	6-Undecanone	-	-	-	-	-	3.69±0.21	-	-	-
21	2-Undecanone	-	-	-	-	-	-	13.9±4.31	-	-
22	3-Pentadecanone	-	-	-	-	-	-	-	8.85±0.72	-
23	9, 9-Dimethoxybicyclo [3.3.1] nona-2,4-dione	-	-	-	-	-	-	-	-	1.83±0.28
24	(Z)-6, 10-Dimethyl-5, 9-undecadien-2-one	-	-	-	-	-	6.39±0.85	5.32±0.61	6.34±0.86	2.22±0.28
25	N-[4-Bromo-n-butyl]-2-piperidinone	-	-	-	-	-	4.68±0.11	17.75±1.46	24.86±1.94	35.39±0.13
					Acids（28）					
1	Pentanoic acid	-	-	-	-	-	-	12.81±0.38	-	-
2	2-Propylmalonic acid	-	-	-	-	-	7.39±1.01	8.47±1.37	19.36±1.67	28.95±0.99
3	1, 2, 4-Benzenetricarboxylic acid	-	-	-	-	-	-	4.36±1.03	3.26±0.27	3.01±0.77
4	2-Methylbutyric acid	-	-	-	-	-	3.72±0.77	-	-	-
5	3-Methylbutyric acid	-	-	-	-	-	-	-	-	54.81±24.73
6	2-Octynoic acid	-	-	-	-	-	-	-	-	2.51±0.18
7	(R)-Methyl ester 4-(1, 1-dimethyletho	-	-	-	-	-	-	-	-	8.26±0.13

Compounds	Name	Absolute Content (μg/kg)								
		HT1	HT2	HT3	HT4	HT5	HT6	HT7	HT8	HT9
	xy)-3-hydroxy-butanoic acid									
8	Cyclohexane butanoic acid	-	-	-	-	-	15.10±1.52	20.76±1.10	43.56±1.15	3.42±0.49
9	2-Decanynoic acid	-	-	-	-	-	20.40±0.87	30.28±1.09	37.33±9.66	40.24±1.04
10	Hexanoic acid	-	42.04±12.74	295.57±256.79	525.84±172.05	268.35±102.11	74.26±26.65	9.40±0.80	47.42±0.12	21.06±1.65
11	Benzyl oxy tridecanoic acid	-	-	-	-	-	102.94±0.71	-	-	-
12	Hexanoyl chloride	-	-	-	-	-	-	32.05±20.07	46.29±5.02	57.31±0.80
13	Oxalic acid, dodecyl 2-methylphenyl ester	-	-	-	-	-	2.74±0.20	-	6.40±0.33	-
14	Cis-2-octenoic acid	-	169.21±36.55	-	-	-	132.81±9.15	-	105.63±9.34	153.10±42.41
15	9-Decen-1-yl ester hexanoic acid	-	-	-	-	-	-	-	8.86±1.11	2.31±0.32
16	(E)-5-Hydroxy-3, 4, 4-trimethyl-2-hexenoic acid	-	-	-	-	-	-	18.42±0.30	19.27±0.38	20.70±6.77
17	Nonyl ester 2-chloro-Acetic acid	-	-	-	-	-	16.24±1.68	-	-	-
18	7-Oxooctanoic acid	-	-	-	-	-	17.27±1.77	-	27.84±14.88	-
19	8-Nonenoic acid	-	-	-	-	-	-	-	-	61.62±1.65
20	Octanoic acid	-	31.70±0.27	93.18±12.71	115.93±20.21	114.63±41.16	16.25±1.81	7.65±0.24	8.43±0.96	55.10±7.91
21	Undecylenic acid	-	-	-	-	-	-	-	-	4.60±0.25
22	1-Octadecanesulphonyl chloride	-	-	-	-	-	-	3.53±0.16	-	-
23	trans-2-undecenoic acid	-	-	-	-	-	-	-	-	2.44±0.26

Compounds	Name	Absolute Content (μg/kg)								
		HT1	HT2	HT3	HT4	HT5	HT6	HT7	HT8	HT9
24	Nonanoic acid	-	-	61.55±5.87	93.4±1.84	47.14±15.89	3.98±0.21	23.74±0.66	10.17±0.57	11.46±0.68
25	3-Decenoic acid	-	-	-	-	-	-	6.46±0.16	12.55±1.02	7.45±0.87
26	Nonanoyl chloride	-	-	-	-	-	-	10.12±0.57	5.52±0.35	3.92±2.87
27	n-Decanoic acid	-	-	-	-	48.63±2.85	-	22.26±0.46	15.34±0.54	110.22±37.78
28	Undecanoic acid	-	-	-	-	-	-	-	-	2.94±0.89
					Esters（33）					
1	1, 2-Propanediol diformate	-	-	-	-	-	-	-	-	5.96±3.62
2	Formic acid, hexyl ester	-	-	-	-	-	-	-	-	67.17±1.61
3	phenethyl pivalate	-	-	-	-	-	-	11.30±0.56	-	-
4	Benzoate 3-methyl-2-Buten-1-ol	-	176.30±9.95	-	-	-	-	-	-	-
5	n-Caproic acid vinyl ester	-	52.78±27.91	-	-	-	25.01±1.40	43.81±18.29	23.28±2.31	20.15±2.55
6	1, 5-Dimethyl-1-vinyl-4-hexenyl butyrate	-	-	-	-	-	-	-	-	171.26±11.4
7	Carbobenzoxyhydrazide	-	-	-	-	-	8.85±1.48	60.37±1.98	130.21±7.19	125.87±13.65
8	sec-Butyl nitrite	-	-	-	-	-	-	-	-	3.25±0.31
9	4-Hexanolide	-	-	-	-	29.83±1.99	10.37±2.99	4.49±0.31	8.21±0.60	11.54±7.27
10	n-Caproic acid vinyl ester	-	-	-	-	815.28±23.59	187.94±9.31	198.27±7.91	207.16±22.22	54.28±1.99
11	p-Nitrophenyl hexanoate	-	-	-	-	-	-	-	-	74.81±1.50
12	Octyl chloroformate	-	-	-	-	-	-	-	-	42.56±1.90
13	1-octyl formate	-	326.46±14.69	178.68±13.80	308.92±51.45	144.32±12.32	197.33±8.28	249.92±42.48	253.13±31.21	46.84±0.70
14	3-methylpheny	-	-	-	-	-	-	5.19±0.25	-	-

Compounds	Name	Absolute Content (μg/kg)								
		HT1	HT2	HT3	HT4	HT5	HT6	HT7	HT8	HT9
15	l estermethyl carbamic acid cis-3, 3, 5-trimethyl-acetate	-	-	-	-	-	-	35.51±0.59	-	-
16	Cyclohexanol Nonyl acetate	-	-	-	-	-	-	-	-	5.02±0.03
17	3, 7-Dimethyloctyl ethylphosphonofluoridoate	-	-	-	-	-	3.74±0.47	-	-	-
18	5-Butyldihydro-2(3H)-furanone	-	-	-	-	27.40±5.88	-	-	19.05±1.40	-
19	Vinyl caprylate	-	-	-	72.49±23.93	-	-	-	-	-
20	Carbamodithioic acid, N, N-diethyl-, methyl ester	20.64±2.42	-	-	-	-	-	-	-	-
21	Z-10-Tetradecen-1-ol acetate	-	-	-	-	-	-	-	-	2.94±0.34
22	Oxalic acid, allyl pentadecyl ester	-	-	-	-	-	2.29±0.11	5.64±1.98	6.58±2.67	2.46±0.71
23	Carbonic acid, undecyl vinyl ester	-	-	-	-	-	-	3.48±0.22	-	-
24	Sulfurous acid, dodecyl 2-propyl ester	-	-	-	-	-	-	4.68±0.37	-	-
25	Sulfurous acid, pentyl tridecyl ester	-	-	-	-	-	-	4.45±0.46	-	-
26	Sulfurous acid, 2-propyl tetradecyl ester	-	-	-	-	-	2.71±0.23	7.56±0.12	-	-
27	Sulfurous acid, 2-	-	-	-	-	-	-	8.53±1.36	-	-

Compounds	Name	Absolute Content (μg/kg)								
		HT1	HT2	HT3	HT4	HT5	HT6	HT7	HT8	HT9
	propyl tridecyl ester									
28	Sulfurous acid, 2-ethylhexyl isohexyl ester	-	-	-	-	-	-	-	-	3.20±0.71
29	Sulfurous acid, hexyl tridecyl ester	-	-	-	-	-	-	3.77±0.19	-	-
30	Oxalic acid, allyl hexadecyl ester	-	-	-	-	-	-	2.63±0.22	-	-
31	Sulfurous acid, hexyl pentadecyl ester	-	-	-	-	-	-	3.26±0.22	-	-
32	Carbonic acid, prop-1-en-2-yl tridecyl ester	-	-	-	-	-	6.19±0.92	6.83±0.23	7.03±0.49	8.26±1.27
33	Carbonic acid, nonyl prop-1-en-2-yl ester	-	-	-	-	-	-	-	-	3.46±0.19
	Alcohols（68）									
1	1-Pentanol	-	-	-	-	225.49±6.72	-	-	-	-
2	[R-(R*, R*)]-2,3-butanediol	-	-	-	-	-	-	-	-	21.78±9.02
3	S-(+)-2-octanol	-	-	-	-	-	6.80±0.22	-	-	-
4	3-Methyl-2-heptanol	-	-	-	-	-	-	-	-	6.24±0.52
5	3-Methyl-1-heptanol	-	-	-	-	-	-	3.51±0.41	-	-
6	(S)-(+)-3-Methyl-2-butanol	-	-	-	-	-	-	-	-	5.52±0.78
7	1-Hexanol	-	48.77±12.42	32.02±2.24	64.41±6.82	25.62±1.83	211.80±30.79	139.45±29.33	67.69±4.90	74.93±1.46
8	4-(1-Methylethyl)-cyclohexanol	-	-	-	-	-	5.25±0.92	5.27±1.00	5.22±0.29	5.71±0.74
9	1-Heptyn-3-ol	-	-	320.41±30.12	425.6±9.09	-	-	-	-	-

Compounds	Name	Absolute Content (μg/kg)								
		HT1	HT2	HT3	HT4	HT5	HT6	HT7	HT8	HT9
10	3, 3, 6-Trimethyl-1, 5-heptadien-4-ol	-	-	70.35±19.97	76.64±52.13	36.18±9.78	20.84±0.58	3.54±1.36	-	6.21±1.46
11	(E)-2-hepten-1-ol	-	-	-	-	-	4.44±0.39	-	-	-
12	1-Heptanol	-	114.19±24.15	108.71±10.84	189.91±33.92	84.76±4.67	122.75±14.40	123.50±14.06	132.23±14.60	-
13	1-Octen-3-ol	-	763.49±240.19	286.50±47.94	466.86±115.60	425.63±53.09	590.36±99.56	272.44±49.84	594.06±38.36	189.61±44.48
14	(1. Alpha., 2. alpha., 5. beta.)- 2-methyl-5-(1-methylethenyl)-cyclohexanol	-	-	-	-	-	6.68±0.20	-	26.13±0.43	-
15	4-Ethylcyclohexanol	-	41.11±3.33	-	-	-	33.66±8.19	-	42.69±6.67	-
16	2-Ethylhexanol	21.58±1.26	-	-	-	-	-	158.49±20.44	-	132.78±18.32
17	2-Propyl-1-pentanol	-	-	-	-	-	-	-	-	180.34±1.68
18	3, 5-Octadien-2-ol	-	-	-	-	71.38±1.09	52.29±1.61	-	-	-
19	(S)-(+)-5-Methyl-1-heptanol	-	-	-	-	-	3.30±1.10	-	-	-
20	Trans-2-pinanol	-	-	-	-	-	-	-	-	9.10±2.47
21	Cyclooctyl alcohol	-	159.25±45.77	-	-	-	136.62±16.71	50.06±8.14	38.02±2.16	14.61±1.07
22	(E)-2-octen-1-ol	-	-	-	-	-	-	-	-	17.01±3.55
23	(Z)-2-nonen-1-ol	-	-	-	110.66±24.81	433.02±10.35	-	-	150.54±0.89	-
24	1-Octanol	-	190.31±5.50	-	-	-	229.20±25.36	-	-	-
25	(1. alpha., 2. beta., 5. alpha.)-2-methyl-5-(1-methylethenyl)-cyclohexanol	-	-	-	-	-	-	4.58±0.23	-	-
26	Nona-3, 5-dien-2-ol	-	-	-	-	-	-	-	15.14±1.14	-

Compounds	Name	Absolute Content (μg/kg)								
		HT1	HT2	HT3	HT4	HT5	HT6	HT7	HT8	HT9
27	5-ethenyltetrahydro-.alpha.,.alpha. -5-trimethyl-, cis-2-Furanmethanol	-	-	-	-	-	8.43±0.55	-	-	-
28	3-Methyl-hepta-1, 6-dien-3-ol	-	155.27±2.62	-	-	246.64±38.06	-	-	-	-
29	4, 5-dimethyl-2-Hepten-3-ol	-	-	748.60±75.32	997.51±117.67	386.26±16.36	46.23±9.26	-	-	-
30	Linalool	-	-	-	-	-	14.54±0.34	99.84±14.49	9.99±1.89	131.84±36.61
31	Isopinocarveol	-	-	-	-	-	-	8.09±0.84	8.28±1.25	8.21±1.41
32	1-(Methylenecyclopropyl)-cyclopentanol	-	-	-	-	-	-	-	6.69±0.94	-
33	(E)-2-Decen-1-ol	-	-	-	-	-	-	-	-	4.12±0.08
34	4, 8-Dimethyl-1-nonanol	-	-	-	-	-	-	-	-	2.98±0.29
35	1, 2-Heptanediol	-	-	-	-	-	-	-	-	4.38±0.26
36	2-Nonen-1-ol	-	-	-	-	-	5.18±0.57	8.27±1.63	12.55±0.96	15.27±0.88
37	4-Methyl-4-nitro-pentanol	-	-	-	-	-	-	-	-	2.32±0.15
38	trans-2-Dodecen-1-ol	-	-	-	-	-	-	3.87±0.10	-	-
39	1, 2, 6-Hexanetriol	-	-	-	-	-	-	-	7.69±1.85	3.58±0.13
40	Terpinen-4-ol	-	-	-	-	-	-	6.75±0.26	-	-
41	1-Octyn-3-ol	-	-	-	-	-	-	-	12.32±0.21	5.98±3.03
42	1-Nonanol	4.30±1.08	-	-	-	-	-	32.53±9.81	-	-
43	E, E-2, 6-Dimethyl-3,	-	-	-	-	-	-	-	-	3.19±0.14

Compounds	Name	Absolute Content (μg/kg)								
		HT1	HT2	HT3	HT4	HT5	HT6	HT7	HT8	HT9
	5, 7-octatriene-2-ol									
44	(2Z)-3-Pentyl-2,4-pentadien-1-ol	-	-	-	-	-	11.69±0.99	18.22±3.76	21.84±7.72	30.12±3.26
45	(R)-(-) -(Z)-14-Methyl-8-hexadecen-1-ol	-	-	-	-	-	-	7.33±0.28	-	-
46	2-Methyl-2-(4-methyl-3-pentenyl)-cyclopropanemethanol	-	-	-	-	-	-	-	-	4.35±0.08
47	(E)-2-Decen-1-ol	-	-	-	-	-	36.87±2.30	-	-	-
48	4-Ethyl-1-octyn-3-ol	-	-	-	-	-	-	4.51±0.22	-	-
49	2-Hydroxy-1,4,4-trimethyl-bicyclo [3.1.0] hexane-6-methanol	-	-	-	-	-	-	-	-	8.86±0.61
50	1-Dodecen-3-ol	-	-	-	-	-	28.77±4.01	-	-	-
51	trans-2-Undecen-1-ol	-	-	-	-	-	-	-	12.34±0.72	-
52	3, 4-Di[1-butenyl]-tetrahydrofuran-2-ol	-	-	-	-	-	-	10.63±4.26	-	11.37±11.59
53	(2Z)-3-Pentyl-2, 4-pentadien-1-ol	-	-	-	-	-	-	-	6.33±0.36	-
54	4, 4, 6-Trimethyl-cyclohex-2-en-1-ol	-	72.33±28.92	132.55±1.24	187.83±35.98	207.67±208.57	35.06±3.78	42.70±12.85	70.22±10.95	21.77±18.81
55	4, 6, 6-Trimethyl-bicyclo [3.1.1] heptan-2-ol	-	-	-	-	-	2.62±0.22	-	-	-
56	Cyclohexanol, 5-methyl-2-	-	-	-	-	-	-	4.81±1.75	-	2.76±1.67

Compounds	Name	Absolute Content (μg/kg)								
		HT1	HT2	HT3	HT4	HT5	HT6	HT7	HT8	HT9
	(1-methylethenyl)-									
57	2-Butyl-1-octanol	-	-	-	-	-	-	10.93±0.11	7.26±0.29	12.65±0.86
58	2-Butyl-2, 7-octadien-1-ol	-	-	-	-	-	21.61±5.04	-	41.59±19.30	22.66±13.92
59	13-Tetradece-11-yn-1-ol	-	-	-	-	-	-	-	14.65±0.61	-
60	E-11, 13-Tetradecadien-1-ol	-	-	-	-	-	6.46±0.22	-	9.79±2.50	-
61	Nerolidol	-	-	-	-	-	-	4.78±0.83	-	2.31±0.78
62	1-Hexadecanol	-	-	-	-	-	-	-	6.16±0.40	-
63	1-Decanol	-	-	-	-	-	2.60±0.41	-	-	-
64	2, 6-Dimethyl-2, 7-octadiene-1, 6-diol	-	-	-	-	-	-	4.72±0.23	-	-
65	E-2-Hexadecacen-1-ol	-	-	-	-	-	-	4.66±0.19	-	2.59±0.09
66	E-11, 13-Tetradecadien-1-ol	-	-	-	-	-	3.50±0.41	-	-	5.84±1.36
67	1-Dodecanol	-	-	-	-	-	7.91±0.70	-	-	-
68	1, 14-Tetradecanediol	-	-	-	-	-	-	4.56±0.16	-	-
					Hydrocarbons（93）					
1	1-Chloro-3-methyl-butane	-	-	-	-	-	39.94±0.57	-	-	-
2	2-Chloro-pentane	-	-	-	-	-	-	24.02±0.41	-	-
3	Toluene	-	-	-	-	-	4.27±0.43	7.69±1.04	10.42±0.94	-
4	Spiro [2.4] hepta-4,6-diene	-	-	-	-	-	-	8.20±0.22	-	-
5	2-(1, 1-dimethylethyl)-3-methyl oxirane	-	301.64±81.61	349.64±69.51	450.55±92.01	245.08±15.19	186.63±15.97	24.45±8.82	182.42±14.19	7.63±3.46

Compounds	Name	Absolute Content (μg/kg)								
		HT1	HT2	HT3	HT4	HT5	HT6	HT7	HT8	HT9
6	1-Chloropentane	-	-	-	-	-	-	61.53±8.13	-	52.53±15.73
7	o-Xylene	-	-	-	-	-	-	-	-	4.53±0.25
8	p-Xylene	-	-	-	-	-	-	3.17±0.26	-	6.52±0.86
9	Cis-1, 3, 5-octatriene	-	-	-	-	-	-	-	-	2.15±0.30
10	(1-Methylbutyl)-oxirane	-	-	-	-	-	-	3.25±0.21	-	-
11	Styrene	-	-	-	-	-	-	12.14±0.71	-	-
12	1, 3, 5, 7-Cyclooctatetraene	-	-	-	-	-	-	7.10±0.75	-	17.74±5.74
13	(3-Methylbutyl)-oxirane	-	-	-	-	-	3.64±0.22	-	-	-
14	1, 2-epoxyheptane	-	-	52.78±2.06	-	-	-	-	-	-
15	1, 2-Epoxyoctane	-	-	54.95±9.90	-	-	-	-	-	-
16	6-Methyl-5-hepten-1-yne	-	-	-	-	-	-	-	-	2.60±0.15
17	1-Isopropylcyclohex-1-ene	-	-	-	-	-	5.00±0.55	-	19.23±5.92	-
18	2-Methyl-4-octyne	-	-	-	-	-	-	-	-	2.67±0.20
19	3-(1-Methylethyl)-cyclohexene	-	-	-	-	-	-	-	20.92±5.90	-
20	2-Chloro-octane	-	146.04±1.59	-	443.10±102.71	214.81±9.84	-	-	-	-
21	2, 2, 4, 6, 6-Pentamethyl-heptane	-	-	-	-	-	-	-	24.01±9.64	-
22	1-Methyl-3-(1-methylethyl)-benzene	-	-	-	-	-	-	4.59±0.40	-	-
23	D-Limonene	-	-	-	-	-	-	43.43±12.05	29.20±1.38	17.34±1.07

Compounds	Name	Absolute Content (μg/kg)								
		HT1	HT2	HT3	HT4	HT5	HT6	HT7	HT8	HT9
24	3-Ethyl-2-methyl-1, 3-hexadiene	-	-	-	-	-	24.80±3.43	23.17±2.19	55.38±5.70	4.09±0.56
25	(Z)-3-ethyl-2-methyl-1, 3-hexadiene	-	-	-	-	-	-	15.45±0.90	7.27±1.26	3.58±0.29
26	2, 4-Dimethyl-1-decene	-	-	-	-	-	-	-	-	3.72±1.73
27	3-Methyl-4-methylene-hexane	-	-	-	-	-	-	3.23±0.08	-	-
28	1, 5-Dimethyl-7-oxabicyclo [4.1.0] heptane	-	38.00±6.97	-	-	-	-	-	-	-
29	1-Nitrohexane	-	-	-	-	-	-	2.63±0.17	-	-
30	1-Chlorononane	-	-	-	-	-	-	-	-	3.55±0.09
31	1, 2:4, 5:9, 10-Triepoxydecane	-	-	-	-	-	-	3.29±0.23	7.59±0.71	2.16±0.56
32	1, 2, 7, 8-Diepoxyoctane	-	-	-	-	-	-	3.30±0.25	-	-
33	2-Methoxy-2-hexene	-	-	-	-	-	-	20.99±1.86	9.83±0.73	-
34	2, 9-Dimethyl-5-decyne	-	-	-	-	-	-	-	59.72±29.44	40.97±8.57
35	2-Nonyne	-	-	-	-	123.23±16.96	-	-	-	-
36	2-Tridecyne	-	-	90.72±17.35	107.19±4.21	-	-	-	-	-
37	1-Nonene	-	-	-	-	-	-	-	-	62.91±2.26
38	Pentyl-cyclopropane	-	-	-	-	-	-	-	-	5.22±1.34
39	2, 2-Dimethyl-3-vinyl-bicyclo [2.2.1] heptane	-	-	-	-	-	-	6.36±0.43	6.91±1.25	6.14±1.27
40	1, 5, 5-Trimethyl-6-	-	-	-	-	-	-	6.37±0.19	5.67±0.13	5.55±0.53

Compounds	Name	Absolute Content (μg/kg)								
		HT1	HT2	HT3	HT4	HT5	HT6	HT7	HT8	HT9
	methylene-cyclohexene									
41	1-Acetoxymethyl-3-isopropenyl-2-methyl-cyclopentane	-	-	-	-	-	-	-	-	7.34±0.49
42	1-(1-Cyclohexen-1-yl)-ethanone	-	-	-	-	-	-	29.91±0.64	-	-
43	3, 4, 5, 6-Tetramethyl-octane	-	-	-	-	-	-	-	-	2.59±0.11
44	(Z)-4-Tridecene	-	-	-	-	-	-	-	-	3.17±0.24
45	2, 2'-(1, 4-butanediyl) bis oxirane	-	-	-	-	-	3.58±0.75	3.98±0.18	-	-
46	3-Acetoxydodecane	-	-	-	-	-	-	4.39±0.22	5.10±0.36	5.62±0.36
47	(1S, 2R, 4R, 7R)-4-Isopropyl-7-methyl-3, 8-dioxatricyclo [5.1.0.02, 4] octane	-	-	-	-	-	-	-	5.50±0.18	-
48	1-Chloro-tetradecane	-	-	-	-	-	-	-	-	2.49±0.28
49	5-Propyl-decane	-	-	-	-	-	8.48±0.29	14.27±0.38	20.65±1.98	23.63±1.84
50	(Z)-3-Undecen-5-yne	-	-	-	-	-	6.83±0.58	-	-	-
51	(E)-5-Tetradecen-3-yne	-	-	-	-	-	-	-	-	3.89±1.38
52	1, 2-15, 16-Diepoxyhexadecane	-	-	-	-	-	-	-	28.21±1.05	-
53	Dodecane	-	53.01±10.53	-	-	30.59±3.35	32.30±9.50	36.31±1.91	60.15±5.79	31.31±8.95
54	1-Tridecyne	-	-	-	-	-	-	8.05±0.68	-	-

Compounds	Name	Absolute Content (μg/kg)								
		HT1	HT2	HT3	HT4	HT5	HT6	HT7	HT8	HT9
55	1-Ethoxy-4, 4-dimethyl-2-pentene	-	-	-	-	-	-	-	-	7.35±0.63
56	1, 2-Epoxy-5-cyclodecene	-	-	-	-	-	3.41±0.16	-	-	-
57	2-Butyl-bicyclo [2.2.1] heptane	-	-	-	-	-	-	7.50±0.12	-	-
58	4-Dodecyne	-	-	-	-	-	4.20±0.32	5.71±1.14	8.02±0.04	10.28±0.72
59	cis-9-Oxabicyclo [6.1.0] nonane	-	-	-	-	-	-	-	-	1.49±0.18
60	1,2-Diazaspiro (2.5) octane	-	-	37.18±1.58	-	-	-	-	-	-
61	1,3-Bis (1, 1-dimethylethyl)-benzene	-	-	-	-	-	-	3.60±0.11	3.53±0.58	3.52±1.58
62	(Z)-5-Tridecene	-	-	-	-	-	-	5.26±0.10	22.03±2.92	25.37±1.26
63	Octyl-oxirane	-	-	-	-	-	-	-	-	3.39±0.24
64	Tridecane	-	96.20±33.04	39.94±2.93	48.00±0.48	37.05±2.67	47.70±4.95	54.73±12.87	53.60±19.84	16.97±9.63
65	4-Ethyl-3-nonen-5-yne	-	-	-	-	-	11.97±2.67	20.72±3.14	27.20±6.42	30.17±2.26
66	1, 2, 3, 6-tetramethyl-Bicyclo [2.2.2] octane	-	-	-	-	-	5.28±0.22	5.46±0.27	6.06±0.34	7.22±1.26
67	1-Octadecyne	-	-	-	-	-	-	5.03±0.66	10.65±1.27	48.97±10.03
68	3-Methyl-tridecane	-	-	-	-	-	20.13±0.79	20.86±0.38	22.66±1.00	23.18±0.16
69	2-Bromo dodecane	-	-	-	-	-	12.57±0.13	17.11±3.15	28.95±6.17	8.05±1.24
70	Tridecane, 3-methylene-	-	-	-	-	-	-	-	5.70±0.99	-
71	(Z)-6-Tridecene	-	-	-	-	-	3.21±0.21	-	-	-
72	(E)-5-Eicosene	-	-	-	-	-	-	9.75±0.11	-	-
73	Cyclododecane	-	-	-	-	-	-	8.64±0.66	-	-
74	(Z)-4-Tetradecene	-	-	-	-	-	-	6.24±0.35	-	-

Compounds	Name	Absolute Content (μg/kg)								
		HT1	HT2	HT3	HT4	HT5	HT6	HT7	HT8	HT9
75	6-Methyl-tridecane	-	-	33.96±1.84	-	104.68±92.99	83.21±4.53	4.79±0.49	83.79±23.04	34.26±1.83
76	Tetradecane	-	62.11±23.96	-	-	-	-	72.78±13.60	-	-
77	(E)-3-Eicosene	-	-	-	-	-	-	4.98±0.68	-	-
78	Tetradecyl-oxirane	-	-	-	-	-	3.33±0.27	-	-	-
79	1-Chloro-dodecane	-	-	-	-	-	-	3.53±0.27	-	-
80	7-Tetradecene	-	-	-	-	-	-	-	-	2.15±0.43
81	1-Pentadecene	-	-	-	-	-	-	19.83±5.89	-	-
82	1-Tridecene	-	-	-	-	-	-	-	23.87±8.48	-
83	(E)-9-Octadecene	-	-	-	-	-	-	-	22.41±1.31	-
84	Pentadecane	-	-	-	-	26.38±2.10	73.85±3.93	87.96±16.03	104.02±29.98	23.81±4.54
85	7-Methyl-pentadecane	-	-	-	-	-	-	7.59±0.40	-	-
86	2, 6, 10-Trimethyl-dodecane	-	-	-	-	-	-	3.75±0.10	-	-
87	3-Methyl-pentadecane	-	-	-	-	-	-	10.69±1.89	-	-
88	(Z)-7-Hexadecene	-	-	-	-	-	2.62±0.26	-	-	-
89	Hexadecane	-	-	-	-	-	10.76±1.11	22.57±1.53	20.33±1.36	21.77±1.27
90	2, 4, 6-Trimethyl-octane	-	-	-	-	-	-	-	-	3.27±0.32
91	5-Propyl-decane	-	-	-	-	-	-	4.63±0.75	-	-
92	Nonadecane	-	-	-	-	-	-	14.35±0.23	-	-
93	Cyclic octaatomic sulfur	-	-	-	-	-	-	5.80±0.91	11.77±1.58	8.80±1.71
Others（36）										
1	Syn-3-methyl-butyl aldoxime	-	-	-	-	-	-	3.69±0.23	3.14±0.36	2.45±0.18
2	Anti-3-methyl-butyl aldoxime	-	-	-	-	-	-	-	-	6.17±3.64

Compounds	Name	Absolute Content (μg/kg)								
		HT1	HT2	HT3	HT4	HT5	HT6	HT7	HT8	HT9
3	(E)-3-Pyridinecarbaldehyde O-acetyl oxime	-	-	-	-	-	-	-	7.18±0.64	-
4	4, 6-Dimethyl-pyrimidine	-	-	-	-	-	-	-	-	5.50±1.88
5	2, 6-Dimethyl-pyrazine	-	-	-	-	-	-	-	-	15.68±7.27
6	4-Hydroxyphenylacetamide	-	-	-	-	-	-	4.78±0.66	7.83±0.33	8.35±1.25
7	Paradrine	-	-	-	-	-	-	-	6.56±0.16	-
8	N-succinimidyl benzoate	-	-	-	-	-	106.19±3.62	93.11±3.71	249.54±21.70	71.64±15.32
9	4-Methylene-1-(1-methylethyl)-cyclohexene	-	-	-	-	-	-	-	-	5.25±1.89
10	2-Pentyl-furan	-	85.17±30.14	79.26±3.14	123.51±16.15	92.22±4.00	81.74±0.90	204.61±22.29	176.52±25.69	102.06±22.96
11	(Dimethylaminomethylene)malononitrile	8.51±0.48	-	-	-	-	464.45±18.44	-	-	-
12	2, 6-Dimethyl-4-pyridinamine	-	-	-	-	-	-	-	-	9.22±5.68
13	2-Methylpentanoic anhydride	-	1228.71±460.84	1582.17±215.26	1891.77±446.97	835.46±49.23	822.37±17.73	612.88±34.28	421.27±11.24	39.62±1.76
14	2-Methoxy-phenol	-	-	-	-	-	-	-	-	21.21±0.45
15	Mequinol	-	-	-	-	-	-	-	-	19.00±5.38
16	Inosine	-	-	-	-	-	-	20.36±4.13	20.18±3.18	21.47±4.38
17	2, 6, 6-Trimethyl-bicyclo [3.1.1] hept-3-ylamine	-	-	-	-	-	-	8.60±0.30	3.16±0.32	7.59±0.42

Compounds	Name	Absolute Content (μg/kg)								
		HT1	HT2	HT3	HT4	HT5	HT6	HT7	HT8	HT9
18	2-(3-Methylbutyl)-thiophene	-	-	-	-	-	8.56±0.19	11.46±2.58	8.44±1.19	8.25±1.21
19	2-Pentyl-thiophene	-	-	-	-	-	-	-	11.64±0.15	-
20	5-Hydroxyuracil	-	-	-	-	-	-	5.52±0.33	-	-
21	Creosol	-	-	-	-	-	-	10.69±1.09	-	-
22	2-n-Heptylfuran	-	-	-	-	-	-	10.53±5.49	-	-
23	N-[3-[N-Aziridyl] propylidene]-2-[2-pyridyl] ethylamine	-	-	-	-	-	-	-	29.44±0.96	9.36±2.11
24	2-Propyl-pyridine	-	-	-	-	-	12.93±5.11	-	-	-
25	1-Pentyl-1H-pyrrole	-	-	-	-	-	-	-	-	1.98±0.12
26	3, 6, 6-Trimethyl-2-norpinanol	-	-	-	-	-	7.96±0.28	-	-	-
27	1, 4-Dimethyl-piperazine	-	-	-	-	-	-	-	-	58.81±13.31
28	Anethole	14.74±5.45	-	-	-	-	-	-	-	-
29	Estragole	-	59.95±14.04	47.96±0.48	-	-	-	140.01±9.64	26.53±7.42	92.38±15.56
30	L-Glucose	-	-	69.53±5.22	-	-	-	-	-	-
31	1-(Dodecyloxy)-2-nitrobenzene	-	-	-	-	-	-	-	10.20±0.81	-
32	Nonyl tetradecyl ether	-	-	-	-	-	-	5.51±0.24	-	-
33	Decyl octyl ether	-	-	-	-	-	-	6.48±0.40	-	-
34	Nonanenitrile	-	-	-	-	-	-	-	-	3.76±0.56
35	Undecanenitrile	-	-	-	-	-	-	-	-	3.51±0.36
36	Decanenitrile	-	-	-	-	-	-	-	-	2.70±0.74
